# Developing a clinical translational neuroscience taxonomy for anxiety and mood disorder: protocol for the baseline-follow up Research domain criteria Anxiety and Depression (“RAD”) project

**DOI:** 10.1186/s12888-016-0771-3

**Published:** 2016-03-15

**Authors:** Leanne M. Williams, Andrea N. Goldstein-Piekarski, Nowreen Chowdhry, Katherine A. Grisanzio, Nancy A. Haug, Zoe Samara, Amit Etkin, Ruth O’Hara, Alan F. Schatzberg, Trisha Suppes, Jerome Yesavage

**Affiliations:** Department of Psychiatry and Behavioral Sciences, Stanford University School of Medicine, Stanford, CA 94305 USA; Sierra-Pacific Mental Illness Research, Education, and Clinical Center (MIRECC) Veterans Affairs Palo Alto Health Care System, Palo Alto, CA 94304 USA; Psychology, Palo Alto University, 1791 Arastradero Road, Palo Alto, CA 94304 USA; Veterans Affairs Palo Alto Health Care System, Palo Alto, CA 94304 USA

**Keywords:** Research Domain Criteria (RDoC), Brain circuits, Anxiety, Depression, Brain imaging, Emotion, Cognition

## Abstract

**Background:**

Understanding how brain circuit dysfunctions relate to specific symptoms offers promise for developing a brain-based taxonomy for classifying psychopathology, identifying targets for mechanistic studies and ultimately for guiding treatment choice. The goal of the Research Domain Criteria (RDoC) initiative of the National Institute of Mental Health is to accelerate the development of such neurobiological models of mental disorder independent of traditional diagnostic criteria. In our RDoC Anxiety and Depression (“RAD”) project we focus trans-diagnostically on the spectrum of depression and anxiety psychopathology. Our aims are a) to use brain imaging to define cohesive dimensions defined by dysfunction of circuits involved in reactivity to and regulation of negatively valenced emotional stimulation and in cognitive control, b) to assess the relationships between these dimension and specific symptoms, behavioral performance and the real world capacity to function socially and at work and c) to assess the stability of brain-symptom-behavior-function relationships over time.

**Methods and design:**

Here we present the protocol for the “RAD” project, one of the first RDoC studies to use brain circuit functioning to define new dimensions of psychopathology. The RAD project follows baseline-follow up design. In line with RDoC principles we use a strategy for recruiting all clients who “walk through the door” of a large community mental health clinic as well as the surrounding community. The clinic attends to a broad spectrum of anxiety and mood-related symptoms. Participants are unmedicated and studied at baseline using a standardized battery of functional brain imaging, structural brain imaging and behavioral probes that assay constructs of threat reactivity, threat regulation and cognitive control. The battery also includes self-report measures of anxiety and mood symptoms, and social and occupational functioning. After baseline assessments, therapists in the clinic apply treatment planning as usual. Follow-up assessments are undertaken at 3 months, to establish the reliability of brain–based subgroups over time and to assess whether these subgroups predict real–world functional capacity over time. First enrollment was August 2013, and is ongoing.

**Discussion:**

This project is designed to advance knowledge toward a neural circuit taxonomy for mental disorder. Data will be shared via the RDoC database for dissemination to the scientific community. The clinical translational neuroscience goals of the project are to develop brain-behavior profile reports for each individual participant and to refine these reports with therapist feedback. Reporting of results is expected from December 2016 onward.

**Trial registration:**

ClinicalTrials.gov Identifier: NCT02220309. Registered: August 13, 2014.

## Background

Globally, one in 13 people suffer from clinical anxiety and about one in 21 suffer from clinical depression [1, 2]. Anxiety and depression are the leading causes of disability and lost productivity worldwide [3]. Despite these alarming statistics, we still lack a valid classification system that links underlying neural mechanisms to individual symptoms, real-world functional consequences and the implications for treatment choices. In the current diagnostic system, the Diagnostic and Statistical Manual, fifth edition (DSM-5) [4] the broad categories of anxiety and mood disorders offer a reliable terminology for communicating between clinicians. The DSM-5 (and earlier editions) is not designed to reflect valid categories in terms of underlying neural function. At least 50 % of people have concurrent diagnoses from more than one category of anxiety and mood disorder [5–7]. Due to the heterogeneity of these categories, it is also possible for two people to both be diagnosed with an anxiety disorder but share only one symptom, and the same is the case for depressive disorders. Individuals diagnosed with anxiety and depressive disorders, and their comorbidity, are also commonly treated with the same medications or behavioral therapies [8], reflecting our limited understanding of the distinct underlying mechanisms that could serve as targets for each intervention.

The National Institute of Mental Health has launched the Research Domain Criteria (RDoC) initiative, which is intended to advance the validity of mental disorder classification by incorporating neuroscience [9, 10]. The ultimate goal of the RDOC initiative is to incorporate neuroscience in ways that will bridge the gap between research and clinical decision-making by helping to define cohesive subgroups relevant to treatment selection and to identifying targets for new interventions. The RDoC initiative is aligned with the vision for precision psychiatry [11] and offers an opportunity for an integrative understanding of mood and anxiety disorder [12].

**Fig. 1:**
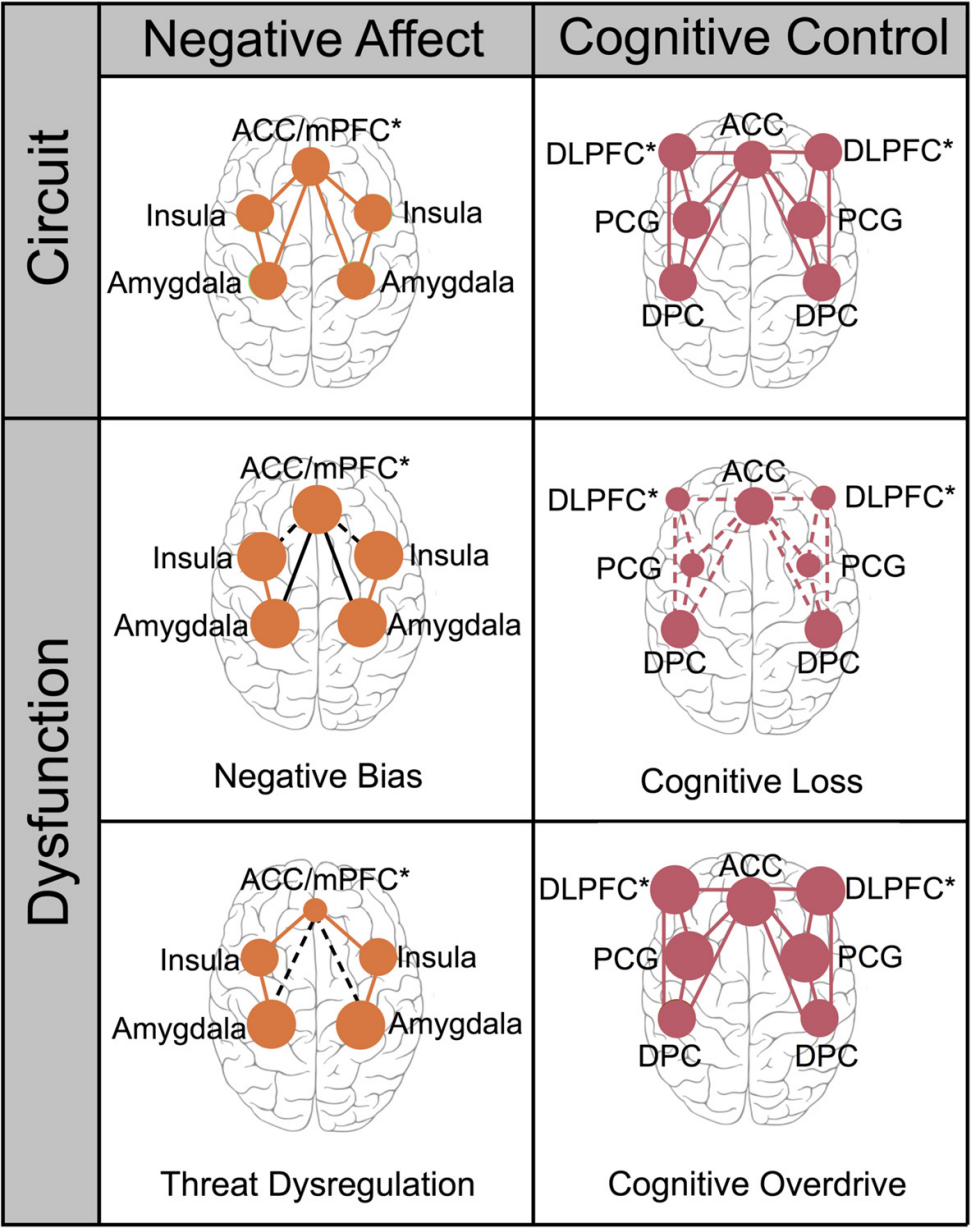
Summary of negative affect and cognitive control circuits and the proposed extremes of dysfunction within these circuits

Supported under the RDoC initiative, the present study is aimed at advancing a brain circuit-based taxonomy relevant to the spectrum of anxiety and depression. Brain imaging research in anxiety and depression has identified core nodes of large-scale circuits that are dysfunctional in these disorders. Here our primary focus is on two large-scale neural circuits found to be relevant to the phenotypes of anxiety and associated mood disorders [11]: the “negative affect” circuit and the “cognitive control” circuit (Fig. 1).

### “Negative affect” circuit

The negative affect circuit is engaged by negatively valenced stimuli and comprises subcortical nodes in the amygdala, brainstem regions, hippocampus and insula and both dorsal and ventral prefrontal nodes – dorsal medial prefrontal cortex (dmPFC) and dorsal ACC connections as well as ventral mPFC (vMPFC) and ventral (subgenual and pregenual)-rostral ACC connections [13, 14]. Dorsal/rostral nodes have been preferentially implicated in appraisal and expression of emotion and may be considered an “aversive amplification” sub-network [14] whereas the ventral nodes are implicated in automatic regulation of negative emotion [13, 15]. These sub-networks may be engaged even in the absence of conscious sensory awareness, via direct brainstem inputs [16] (for meta-analysis [13]). In light of their commonly observed co-activation [13], the negative affect circuit could subserve the perception of negative emotion cues and the salience circuit, the arousal aspects of feeling these emotions.

### “Cognitive control” circuit

The “cognitive control” circuit comprises the dorsolateral prefrontal cortex (DLPFC), anterior cingulate cortex (ACC), dorsal parietal cortex (DPC) and precentral gyrus [11]. Together these regions and their interconnectivity are implicated in the support of higher cognitive functions such as working memory and selective attention (for meta-analysis [17]), evidence from convergent neuroimaging methods [18]). Under task-specific demands the cognitive control circuit is implicated in cognitive flexibility [19].

In the current study we use these circuits as the independent (rather than dependent) variable to parse neural circuit-based types of dysfunction in a trans-diagnostic manner. We conceptualize types that represent extremes along dimensions of dysfunction within these circuits and their interactions.

### Proposed types of dsyfunction in the “Negative affect” circuit

We hypothesize that negative affect circuit dsyfunctions will be expressed as hyper-reactivity in the bottom-up innervation of the amygdala and insula at one extreme and loss of top-down regulation of emotion-elicited reactivity at the other extreme (Fig. 1). These putative biotypes may contribute to subjectively experienced phenotypes of “negative bias” and “threat dysregulation” [11].

Amygdala hyper-reactivity elicited by non-conscious processing of masked threat stimuli has been reported in current depressive disorder (for review [20]), generalized anxiety disorder [21], generalized social phobia/anxiety disorder [21–23], specific phobia [24] and panic disorder [21, 24]. Mood-congruent hyper-reactivity of the amygdala has also been observed in response to sad faces [25, 26]. By contrast, the opposite finding of *reduced* amygdala activation for positive expressions has been observed in depressed people [27, 28]. There are also findings of amygdala hypo- (rather than hyper-) reactivity for threat stimuli, which may characterize unmedicated participants who go on to respond to typical first-line antidepressants [27, 29].

Correspondingly, ACC hypo-activation during the processing of threat stimuli has been observed in generalized anxiety disorder [15, 30] and generalized social anxiety [30]. Reduced connectivity between the amygdala subgenual/ventral ACC has been observed during the processing of masked threat stimuli in unmedicated MDD [31], generalized social anxiety disorder [32] and generalized anxiety disorder [15].

### Proposed types of dsyfunction in the “Cognitive Control” circuit

We hypothesize that cognitive control circuit dsyfunctions may also be expressed as hyper-reactivity at one extreme and hypo-activation reflecting loss of top-down control at the other extreme. These putative biotypes may contribute to subjectively experienced phenotypes of “cognitive overdrive” and “cognitive dyscontrol” [11] (Fig. 1).

Depressed people show hyper-activation of the DLPFC during working memory and executive function tasks, which may reflect an attempt at compensation to retain normal behavior (Fig. 1). DLPFC hyper-activation has been observed during tasks with an increasing cognitive demand, but in the absence of performance deficits, in, medicated MDD [33] and unmedicated MDD [34]. Hyper-activation of the ACC has also been observed in MDD when participants are performing similarly to controls (rostral ACC [33]) and this effect persists after remission.

Dysfunction of the cognitive control circuit may be elicited by tasks requiring effortful selective processing of relevant while inhibiting irrelevant stimuli, and suggests a “cognitive loss” type (Fig. 1). Hypo-activation of the DLPFC and dorsal anterior cingulate cortex (dACC) has been observed across diagnoses, including depression [35–37] and social anxiety [38]. Correspondingly, depression has been associated with a loss of functional DLPFC-dACC connectivity when cognitive control is required [36]. Hypo-activity in these nodes of the cognitive control circuit has been found to persist after recovery in adult and later-life depression [39] suggesting that a “cognitive loss” type may have a trait-like status.

### Linking brain circuit dysfunction to symptom profiles

It is not yet known how dysfunctions involving the negative affect and cognitive control circuits relate to specific features of anxiety and mood symptoms. Here we assess multiple features of anxiety, depression and general distress, encompassing constructs of “anxious arousal”, “apprehensive expectations” (and rumination) and anhedonia. Paralleling the findings for brain imaging of neural circuits, a wide range of disorders share common symptoms. For example, multiple anxiety disorders (e.g., specific phobia, panic and social anxiety) share symptoms of anxious arousal (implicating dysfunctions in bottom-up threat reactivity), while other general and trauma-related anxiety disorders (GAD and PTSD) are accompanied by more cognitive experiences of anxiety, such as apprehensive expectations (implicating lack of emotion regulation) [40, 41]. Data-driven techniques such as factor analysis may be useful in identifying specific dimensions of symptoms that cut across traditional diagnoses [42].

The RDoC Anxiety and Depression (“RAD”^1^) study is supported by the NIMH RDoC initiative, and designed to make progress toward a cohesive brain-based taxonomy relevant to the spectrum of anxiety and associated mood disorders and associated impairments in behavior and real-world function.

## Research objectives

### Primary objectives

1. To use brain imaging to identify cohesive transdiagnostic dimensions of dysfunction in the “negative affect” circuit for threat reactivity and regulation, and associated dysfunctions in the “cognitive control” circuit. We will also assess interactions between these brain circuit dysfunctions.
2. To assess whether brain circuit-defined dysfunctions relate to the severity of specific anxiety and mood symptoms, such as anxious arousal and apprehensive expectations.
3. To assess whether these brain circuit-defined dysfunctions also relate in a cohesive way to behavioral performance and to real world functions related to burden of illness, specifically social functioning, quality of life and work productivity.

### Secondary objective

To assess if the brain circuit-defined dimensions predict change in symptoms and real world functions over time.

## Ethical approval

The Institutional Review Boards of Stanford University and Palo Alto University have approved this protocol. Informed written consent and permission to publish any direct quotes from interviews will be obtained from each participant. Consent will also be obtained to video-record feedback sessions to be used for training purposes.

## Methods and design

We use a baseline-follow up design. Intensive assessments for all measures of interest are undertaken at the baseline session and after 12 weeks participants are re-assessed at follow-up on the symptom and functional outcome measures (Table 1). We control for medication-free status at the baseline session and record in an observational (rather than experimental) manner any interventions that participants were exposed to in the 12-week period between baseline and follow up. Because this was not an intervention study, but rather an investigation of the natural stability of brain-behavior-symptom relationships over time we do not control treatment type or intensity. Our clinical center partners use a structured treatment planning approach based on integrative psychotherapy principles with pharmacological augmentation and all participants recruited from the center are currently receiving individual therapy.

**Table 1:** A summary of the test-retest design and type of data collected at each assessment time point

Screening	Baseline	Follow-up
Self-reported symptoms	Structured Clinical interview	
Contraindications for medication	Brain imaging of circuits	
	Behavioral performance	
Contraindications for brain imaging	Self-reported symptoms	Self-reported symptoms
Other contraindications for study participation	Self-reported and observer-rated coping, social and occupational function	Self-reported and observer-rated coping, social and occupational function

Participants are free of antidepressant medications and other medications that could impact the brain imaging assessments.

Consistent with the goal of RDoC and characterizing brain imaging-derived (rather than diagnostic) constructs, screening and exclusion criteria are kept to a minimum. We do not exclude for cognitive indicators of potential mild cognitive impairment (MCI) given that such cognitive deficits in many patients are associated with their anxiety and mood symptoms but are not indicative of neurodegeneration (for review; [43]). These indicators are recorded for inclusion as covariates in analyses.

For participants who meet inclusion criteria and are not screened out by exclusion criteria we schedule a testing site visit. At the testing site visit brain imaging, behavioral and self-report/observer-rated assessments are completed (Table 1).

### Recruitment

We aim to enroll 160 participants who comprise a clinical-community sample. Enrollment commenced in August 2013, and we anticipate enrollment for baseline assessments will be completed in 2017.

We are enrolling patients from the Gronowski Center (GC), a community mental health training clinic, and individuals from the immediate surrounding community. The target of 160 ensures sufficient statistical power and coverage of the spectrum of anxiety and associated mood symptoms. Our power calculation is designed to test for an anticipated regression model with a medium effect size; approximately .5 (or f^2^ = .0625). With an alpha level of .05, a power level of .80, two primary predictors (our R0Is) and four additional predictors (behavioral and questionnaire measures) we require at least 158 participants. We have anticipated screening at least 3 times the target number in order to achieve a total enrolled sample of 160.

We are not using psychiatric diagnosis as inclusion criteria. We include participants spanning the dimension from healthy through sufficiently severe to meet diagnostic criteria.

To demonstrate the spread of coverage from our recruitment strategy, we record diagnosis (or absence of diagnosis), but do not use this information for primary analyses. Based on prevalence data from the existing GC records, we have anticipated the following spectrum of diagnoses: social anxiety disorder (6 %), generalized anxiety disorder (13 %), panic disorder (3 %), agoraphobia or specific phobia (1 %), post-traumatic stress disorder (8 %), obsessive compulsive disorder (4 %), major depressive disorder (26 %), persistent depressive disorder (dysthymia) (12 %), bipolar disorder 1 (5 %), other specified or unspecified depression or anxiety (5 %).

### Inclusion and exclusion criteria

Inclusion criteria are: i) age (18+ years) to focus on the adult brain, ii) fluent and literate in English in order to understand task instructions, and iii) currently reporting distress from anxiety and related mood symptoms. Exclusion criteria are: i) current or lifetime experience of frank psychosis and/or mania, because the circuit dysfunctions associated with such phenomenology might obscure interpretation of anxiety and mood-related circuit dysfunctions, ii) presence of suicidal intent representing imminent risk as indicated during screening and on-site assessments, iii) medical condition or neurological disorder that could impact brain imaging data and render images difficult to interpret, iv) history of physical brain injury or blow to the head resulting in loss of consciousness greater than five minutes and which in the judgment of investigators could interfere with interpretation of brain imaging assessments, and v) severe impediment to vision, hearing and/or hand movement, likely to interfere with the ability to complete the assessments, or follow the instructions.

We record information about comorbid conditions that are not part of the exclusion criteria, including substance use and other general medical conditions. In regard to substance use disorders, we accept participants who are using alcohol and substances as a comorbid behavior given the emphasis on recruiting a representative sample. A NIH Certificate of Confidentiality that has been granted to the principal investigator protects participants.

### Screening

Participants are screened over the phone during a call that lasts about 15 min. Oral consent is taken, and study details are explained. Questions about participant demographics and inclusion and exclusion criteria are asked. Inclusion criteria questions include being at least 18 years of age, having recent symptoms of anxiety or depression, and speaking English. Exclusion criteria questions include recently taking psychiatric medications, traumatic brain injuries, MRI contraindications (e.g., implanted devices, pregnancy, claustrophobia, ferromagnetic material in the body), and psychosis. At the end of the screening interview, a determination of the participant’s eligibility is made with the approval of the principal investigator.

### Baseline visit

At the baseline visit we undertake a traditional clinical interview, using the Mini-International Neuropsychiatric Interview (MINI-Plus) [44]. We also assess demographics (including age, sex, education and handedness using the Edinburgh Handedness Inventory), and prior and current medical status. Detailed assessments targeting our primary aims are also undertaken at the unmedicated baseline visit, and Table 2 provides a tabular summary of these measures.

**Table 2:** A summary of the measures used in the trial to assess circuit function, behavior and self-report and observer rated symptoms and real-world function

fMRI Paradigms	Behavioral Tasks	Self and Observer Report	
Negative Affect Circuit		Clinical Measures	Real World Function
Masked Facial Expressions of Threat	Threat Identification and Bias	Symptoms of anxiety and negative mood:	Coping:
Threat Conflict Regulation	Threat Conflict Adaptation	BAI, PSWQ, BDI, QIDS-SR, DASS, MASQ	COPE
			Emotion Regulation:
			ERQ
Cognitive Control Circuit		Symptoms of associated traumatic stress, impulsivity, and substance use:	Social and Occupational Function:
Go-NoGo	Go-NoGo	PCL-C, BIS, FTND, DSM 5 AUD, DSM 5 SUD, AUDIT, CUDIT, NM-ASSIST	SOFAS
			Quality of Life:
			WHOQoL, SWLS
N-Back Continuous Performance	Digit Span	Trauma-related risk:	Work productivity:
	Verbal Learning and Memory	ELSQ	HPQ

*Abbreviations*: *BAI* Beck Anxiety Inventory, *PSWQ* Penn State Worry Questionnaire, *BDI* Beck Depression Inventory, *QIDS-SR* Quick Inventory of Depressive Symptomatology-Self Report, *DASS* depression anxiety stress scales, *MASQ* Mood and Anxiety Symptom Questionnaire, *PCL-C* Posttraumatic Stress Disorder CheckList-Civilian Version, *BIS* Barratt Impulsivity Scale, *FTND* Fagerstrom Test for Nicotine Dependence, *DSM 5 AUD* DSM 5 Alcohol Use Disorder, *AUDIT* alcohol use disorders identification test, *DSM 5 SUD* DSM 5 substance use disorder, *NM-ASSIST* NIDA-Modified Alcohol, Smoking and Substance Involvement Screening Test, *CUDIT* Cannabis Use Disorder Identification Test, *ELSQ* Early Life Stress Questionnaire, *COPE* Brief COPE, *ERQ* Emotion Regulation Questionnaire, *SOFAS* Social and Occupational Functional Assessment Scale, *WHOQOL* World Health Organization Quality of Life Scale, *SWLS* satisfaction with life scale, *HPQ* World Health Organization Health and Work Performance Questionnaire

### 1. Clinical interview, medical history and demographics

At baseline the MINI-Plus [44] is used to assess DSM-IV criteria for anxiety and mood disorder and to confirm exclusions due to psychosis and/or clinically significant mania. To ensure currency we also apply DSM-5 criteria to the information gathered at interview. Research personnel also gather comprehensive information on past and current medical history and additional sociodemographic data.

### 2. Brain imaging

Participants undergo a brain scan protocol that will include a battery of previously established standardized paradigms [45].

#### Paradigms

##### i) Viewing of threat faces

We selected the masked viewing of threat faces paradigm because it  reliably engages negative affect circuitry, is grounded in proposed mechanisms of anxiety, and has been well established by the investigators. In the masked condition this paradigm probes automatic bottom-up activation of the negative affect circuit [16, 46, 47]. Stimuli are from a standardized series of facial expressions of threat-related emotions (fear, anger), loss related emotions (sadness) and reward-related emotions (happiness), along with neutral, modified such that the eyes are presented in the central position of the image [48]. Stimuli are presented for 16.7 ms, determined to be below the sensory threshold for conscious identification of emotion, followed immediately by a neutral face perceptual mask for 150 ms and an interstimulus interval of 1233.3 ms. Mask stimuli are offset by 1° in the direction of each diagonal, randomly, in order to control for the potential effects of priming due to the perceptual difference between emotion-neutral and neutral-neutral target-mask pairs. Using a blocked design, stimuli will be grouped with eight faces expressing the same emotion per block and repeated 5 times in a pseudorandom order [45]. Using behavioral psychophysical testing, we have shown that when faces in this paradigm are presented at or below 20 ms, they meet signal detection criteria for being at the subliminal threshold for detection, such that individual participants cannot detect the presence of the face nor discriminate the facial expression [48].

To provide a positive control for the masked condition we also present the same 240 standardized facial expressions described above in an explicit conscious perception condition. In this condition stimuli will be presented for 500 ms, with an interstimulus interval of 750 ms, also in a blocked design. The stimulus duration of 500 ms was based on evidence that this is sufficient time to allow for conscious elaborative processing of the emotion stimulus. Conscious discrimination of emotion is consistently above chance (and close to 100 %) at durations ≥330 msec [48] and facial expressions of emotion consistently elicit a contagious effect of experiencing the emotion signaled by the stimulus at durations of 500 ms [49]. Stimulus onset asynchronies are standardized at 1250 ms across both masked and explicit perception paradigms. No specific behavioral responses were required during scanning because of the inclusion of subliminal presentations and our aim to isolate activation elicited by emotion stimuli independent of behavioral task demands. A large meta- analysis of 385 studies has shown passive processing is associated with a higher probability of activation than an active task [50]. We created a context for participants to continuously view the faces by instructing them that they would be asked post-scan questions about these faces. We will control for active attention to the face stimuli by monitoring alertness with an eye tracking system.

##### ii) Threat conflict regulation paradigm

We selected the threat conflict paradigm because it also reliably engages negative affect circuitry, in this for top-down regulation of emotional reactivity, and is also well established by the investigators [15, 51].

In the threat regulation task participants are presented with a total of 148 happy or fearful facial expressions [52], while ignoring an overlying word labeling the expression (“FEAR” or “HAPPY”). The word either matches the facial expression (congruent) or conflicts with it (incongruent). Each stimulus will be presented for 1000 ms with a variable interstimulus interval (mean: 4000; range: 3000-5000 ms). Stimuli will be presented in a psudorandom order [51].

##### iii) Go-NoGo paradigm

We use the Go-NoGo paradigm that has been established as a robust probe of the cognitive control circuit [45, 53, 54]. The Go-NoGo paradigm is used to assess impulsivity (automatically-generated ‘Go’ responses) versus inhibition (‘NoGo’ responses). In the ‘Go’ trials, participants are required to “press” on GREEN stimuli (the word “press”), while in the ‘NoGo’ trials; participants withhold presses on RED stimuli. Stimuli are presented for 500 ms each with an inter-stimulus interval of 750 ms. Participants are asked to respond via button press as quickly as possible to the Go stimuli and inhibit their response for the NoGo stimuli. Reaction times and number of errors on task are used to evaluate task performance. The design of this paradigm allows for event-related analysis. The probability of NoGo stimuli is .33. There are a total of 180 Go and 60 NoGo stimuli presented in a pseudorandom order with a constraint to ensure that NoGo stimuli are not repeated more than 3 times in a row.

##### iii) N-back Continuous Performance Test

As a complement to the Go-NoGo paradigm used to assay cognitive control functions we also use an n-back Continuous Performance Test (CPT) to assess sustained attention and selective working memory updating [45]. In the “sustained attention” target trials, participants press a button when the same letter appears twice in a row (a ‘1-back’ design). In the “working memory updating” trials participants are required to continually update the contents of working memory in case of a target. In total there are 120 letters (B, C, D or G) presented sequentially, including 20 sustained attention targets in yellow, 60 working memory updating stimuli in yellow and 20 addition “to be ignored” letter in white which were intended to provide a perceptual baseline condition. Stimuli are presented for 200 ms each with an inter-stimulus interval of 2300 ms. Presentation was pseudorandom, ensuring there were no consecutive target tones. One fMRI volume per stimulus was acquired.

#### Acquisition and quantification

##### i) Functional scans

For each paradigm, blood oxygenation level-dependent contrast functional images are acquired with echo-planar T2*-weighted imaging using 3.0 Tesla GE Signa HDx scanner (GE Healthcare, Milwaukee, Wisconsin) with a 32-channel head coil. Each whole brain volume will consist of 45 interleaved 3 mm thick axial/oblique slices (74 x 74 matrix; TR, 2000 ms; TE, 27.5 ms; size, 3 x 3 x 3 mm; FOV, 222 mm; flip angle, 77°). 154 volumes will be acquired over 5.03 min and 8 s for all paradigms but the emotion regulation task. For the emotion regulation task a total of 397 volumes will be collected over 13 min 14 s. Three dummy scans are acquired at the start of each acquisition.

Preprocessing and data analysis is performed using Statistical Parametric Mapping software implemented in Matlab (SPM8; Wellcome Department of Cognitive Neurology) in a manner similar to that of our prior publications [27, 45]. Specifically, Motion correction is performed by realigning and unwarping the fMRI images to the first image of each task run. Following realignment and unwarping, quality control diagnostics are completed on the time series data for each run. Images are normalized to the stereotactic space of the Montreal Neurological Institute (MNI) template [55], T1- weighted data are normalized to standard space using the FMRIB nonlinear registration tool and the fMRI EPI data are coregistered to the T1 data using FMRIB linear registration tool. Normalization warps from these two steps are stored for use in functional to standard space transformations. Global signal is estimated using a eroded mask within the ventricles and white matter and is removed from the motion-corrected fMRI time series. fMRI data are smoothed using an 8 mm Gaussian kernel and high-pass filtered using a cutoff period of 128 s.

To define circuit-based constructs we focus first on specific nodes in the circuits of interest using a region of interest (ROI) approach, established previously [56]. With the RO1 approach we identify BOLD-dependent signal change in the defining nodes of the negative affect circuit, including amygdala, insula, ACC/mPFC (ventral and dorsal). Beta values for each ROI are extracted for each subject for regression analyses. We will also use functional connectivity analyses to quantify the functional relationships between regions. Further, exploratory whole brain, voxel-wise analyses are conducted using a significance threshold of p < 0.05 corrected for multiple comparisons. Regions of activation are defined according to the Talairach Atlas. In parallel we study additional regions as part of the exploratory goals of the study.

##### ii) Additional exploratory functional analyses

Since we recognize that the amygdala and ACC are part of more extensive circuits, in the exploratory phase we will also assess activation in other regions, informed by the extant literature and any new developments in the field.

##### iii) Structural scans

A high-resolution T1-weighted structural scan is acquired using a 3D spoiled gradient echo (SPGR) sequence at the end of the imaging session for use in normalization of the fMRI data into standard space. A diffusion scan is also acquired in order to quantify white matter integrity.

### 3. Behavior

#### Computerized tests of behavioral performance

Threat identification and bias: The same faces as shown during fMRI are presented on a computer screen (96 stimuli, 8 different individuals). Identification is recorded by the verbal labeling of the expressions and the reaction time to do so. Implicit priming of reaction time to “old/new” memory recognition of faces, primed by prior exposure to facial expressions of threat versus neutral, to elicit biases to threat using an established protocol [57]. The bias to fear is the reaction time difference (in milliseconds) for priming due to threat minus neutral.

Threat conflict adaption: The Emotional Conflict task generates reliable reaction time interference [15, 51, 58]. We will quantify reaction time for successive trials, indexing adaptive regulation to fear-related conflict.

Go-NoGo: To assess response inhibition we use a previously established task in which participants respond quickly to green stimuli and withhold responses to red stimuli [59].

We will also assess cognitive control using additional behavioral tests of executive function, memory and inhibition, including the following:

Digit span. To assess working memory participants are asked to hold online a span of 2 to 9 digits and then repeat these digits in order.

Verbal learning and memory. To test immediate and delayed recall of verbal information (12-word lists), equivalent to the constructs assessed by the California Verbal Learning and Memory test.

Verbal Interference test: Using previously established tests assessing constructs equivalent to those assessed by the Stroop color/word test [59].

#### Acquisition and quantification

Each of these behavioral tests runs on a desktop computer, and does not rely on keyboard skills. The software used to run the tasks includes standardized task instructions. Psychometric properties have been established for each of these tests, including norms, construct validation, validation against traditional neuropsychological tests tapping equivalent functions, test-retest reliability, and consistency across cultures [59–64]. The tests have been used effectively in patient groups in previous research by the investigators [65–68].

For each test we record accuracy and reaction time via the testing software. These data are logged in a file on the desktop computer under the identification code for each participant.

### 4. Self-report and observer-rated measures

#### Symptoms of anxiety and negative mood

##### i) Beck Anxiety Inventory (BAI)

A 21-item self-report inventory for measuring the severity of common symptoms of anxiety that the participant has had during the past week, such as numbness and tingling, sweating not due to heat, and fear of the worst happening [69].

##### ii) Penn State Worry Questionnaire (PSWQ)

A 16-item questionnaire that assesses items such as “my worries overwhelm me” and is rated on a likert scale, with scores ranging from 1 to 80 [70].

##### iii) Beck Depression Inventory (BDI)

A 21-item, self-report rating inventory that measures symptoms of depression such as hopelessness and irritability, cognitions such as guilt or feelings of being punished, as well as physical symptoms such as fatigue, weight loss, and lack of interest in sex [71].

##### iv) Quick Inventory of Depressive Symptomatology, Self-Report (QIDS-SR)

A 16-item self-report assessment of the nine DSM-IV symptom criteria for major depressive disorder. Individuals rate items as present, mild, moderate severe, and scores range from 0 to 27 [72].

##### v) Depression, Anxiety and Stress Scale (DASS)

The DASS is a 42-item self-report scale that assesses symptoms of depression/anhedonia, anxious arousal and generalized anxiety (stress) that are not tied to a particular diagnosis. The DASS has been normed for use in healthy and patient groups, and validated against other measures of anxiety such as the BAI described above [73].

##### iv) Mood and Anxiety Symptom Questionnaire (MASQ)

A 90-item questionnaire based on the tripartite model of affective disorder which encompasses constructs of anhedonia, anxious arousal and generalized distress, equivalent to the DASS, and which has also been used in healthy and patient groups [74].

#### Symptoms of associated traumatic stress, impulsivity and substance use symptoms

##### i) PTSD CheckList – Civilian Version (PCL-C)

A self-report rating scale for assessing the 17 DSM-IV symptoms of PTSD. This version is a general civilian version that is not linked to a specific event. A total score is computed by adding the 17 items, so that possible scores range from 17 to 85 [75].

##### ii) Barratt Impulsiveness Scale (BIS)

A questionnaire designed to assess the personality/behavioural construct of impulsiveness [76] that may reflect subclinical mania and related experiences.

##### iii) Fagerström Nicotine Dependence Scale (FNDS)

A short 6-item instrument used for assessing the intensity of physical nicotine addiction [77].

##### iv) DSM-5 alcohol use disorder

A 12-item questionnaire assessing alcohol use based on the DSM-5 criteria [4].

##### v) Cannabis Use Disorder Identification Test – Revised (CUDIT-R)

A cannabis misuse screening tool containing 8 items, two from each of the domains of consumption, cannabis problems (abuse), dependence, and psychological features [78].

##### vi) DSM-5 substance abuse disorder

An 11-item questionnaire assessing substance use disorders based on the DSM-5 [4].

##### vii) NIDA-Modified Alcohol, Smoking, and Substance Involvement Screening Test (NM-ASSIST)

A 15-item measure adapted from the World Health Organization (WHO) Alcohol, Smoking and Substance Involvement Screening Test, used to assess prescription medicine and illicit substance use in adults age 18 and older [79].

#### Trauma-related risk factors

Early Life Stress Questionnaire (ELSQ): A 19-item questionnaire used to retrospectively assess exposure to early life stress, by ascertaining whether the participant had experienced physical, emotional, or sexual abuse as well as other traumatic experiences such as sustained bullying, poverty, divorce, illness, or domestic violence. If a stressor was present, the participant identified age of onset (0-12 years of age) [80].

#### Real world function and coping

##### i) Brief COPE

A multidimensional coping inventory to assess the different ways in which people respond to stress [81].

##### ii) Emotion Regulation Questionnaire (ERQ)

A 10-item questionnaire designed to assess individual differences in the habitual use of two emotion regulation strategies: cognitive reappraisal and expressive suppression [82].

##### iii) Social Functioning and Adjustment Scale (SOFAS)

The SOFAS is a derivative of the Global Adjustment Scale and reflects the individual’s level of social and occupational functioning, rated on a scale from between 0 and 100 [83].

##### iv) World Health Organization Quality of Life (WHOQOL) scale

This is a 30-item scale that assesses the psychological, general health, physical and environmental aspects of quality of self-reported quality of life, each assessed out of 100 [84].

##### v) Satisfaction With Life Scale (SWLS)

A short, 5-item instrument designed to measure global cognitive judgments of satisfaction with one’s life [85].

##### vi) Health Productivity Questionnaire (HPQ)

This is a 14-item scale that assesses productivity at work, and relative and absolute levels of absenteeism from work as well as presenteeism (being at work, but unproductive due to the effects of anxiety and depressive symptoms [86].

#### Acquisition and quantification

Each self-report measure is presented in a computerized format. Individual item scores are recorded and then summed automatically according to symptom cluster and scale definitions. The only exception to automated scoring is the SOFAS, which is rated by study coordinators.

### Follow-up outcome measures

At 12-week follow-up, we assess change in/stability of symptoms using the PSWQ, DASS and MASQ, HPQ and WHOQoL.

### Translational outcomes: toward a precision mental health protocol

Participants are offered the opportunity to partake in a brief feedback session (i.e., 30 min) with the study principal investigator, key clinical personnel and their current therapist. The feedback is based on self-report measures such as mood and anxiety symptoms, social and occupational functioning, and behavioral tests of emotion and cognition. A brief clinical summary of the participant’s history is provided to the investigator as contextual information. The investigator discusses the participant’s profile on the measures, describes relevant test performance, and gives recommendations for how this information could be applied to treatment. Anxiety and depression are examined from an RDoC perspective incorporating such dimensions as threat reactivity, threat regulation and cognitive performance; the participant is offered an overview of their experience based on a neural circuitry perspective. The participant and their therapist are given the opportunity to ask questions regarding current symptomatology as related to the neuroimaging and behavioral data. At the conclusion of the feedback session, the investigator exits while the participant and therapist consider the information received. The ultimate goal is to collaborate on incorporating the feedback into the current treatment plan. Treatment outcomes such as symptom reduction and therapy retention will be examined for participants who receive the feedback.

### Data management

All data are de-identified using participant numbers and no session number is identified. Follow-up data is distinguished from baseline visit data with timestamps and distinct variable names.

Source documents will be archived for 7 years beyond study completion, or in accordance with IRB regulations, whichever is longest.

Neuroimaging data are managed using the quality control and data management infrastructure at the Stanford Center for Neurobiological Imaging (CNI). At CNI, the Neurobiological Image Management System (NIMS) has been in use for the past four years. The current version of the software supports standardized format (DICOM) files. NIMS was designed to address key issues in order support the principles of reproducible research by i) enabling data sharing in way that is consistent with current norms in the field of neuroscience and ii) combining imaging data and metadata about subjects in a searchable database system.

For the computerized behavioral tests, the computer registers each response and writes these with time stamps to a log file. For the questionnaires, each self-reported response entered by participants is logged. These data are stored in a PHI-protected database and then integrated with the neuroimaging data.

In accord with the requirements of the project funding award, the data will be shared via the NIMH RDoC database, RDoC-db.

### Statistical analysis

The statistical analysis plan is designed to test specific working hypotheses relevant to each of the primary aims of the study:

1. We will use both a priori approaches to group participants into “high” versus “low” subgroups on each imaging circuit measure, and their combination. We will also use data-derived techniques such as cluster and factor analysis to complement these a priori approaches.
  We will use General Linear Models (GLMs) to compare “high” versus “low” brain circuit-derived subgroups and data-derived clusters on symptom profiles. In correlational models we will assess the relationships between brain imaging dimensions and specific symptom profiles.
2. We will use GLMs to compare brain imaging-derived subgroups/clusters on behavioral performance. We will also test associations between behavior, imaging and symptoms using correlational and mediator/moderator models.
3. We will use GLMs to compare brain imaging-derived subgroups/clusters on real-world function. We will also test associations between function, imaging, behavior and symptoms using correlational and mediator/moderator models.

The statistical analysis plan also addresses the secondary aims of the study:

4. Repeated-measures GLMs, reliability and regression models will be used to assess change (or stability) in symptoms and function at follow-up and to test if imaging at baseline predicts profiles of symptoms and function after 12 weeks.

We will use multiple methods to integrate the data under each aim.

As noted above we will use mediator and moderator approaches to model the relationship between imaging, symptoms, behavior and/or function at baseline and over time.

Second, we will employ multiple methods for implementing data reduction and pattern discovery, depending on the dimensionality and distributions of data domains. For example, we will employ model-based sparse factor analysis [87], a method that has been developed and successfully employed on functional neuroimaging data. We will do so hierarchically, to find simple structure within each domain separately, and then across domains, using Bayesian Belief Nets [88] to obtain directed relationships. We will also examine associations between domains using modern variable selection techniques such as the Elastic Net [89], a combined regression and variable selection methodology that is capable of handling large numbers of correlated explanatory variables. Finally, we will use techniques such as canonical correlation analysis to determine linear combinations of variables in given domains (e.g., neuroimaging) that maximally relate to variables in other domains (e.g., behavioral or symptoms).

### Power calculation

Power is estimated for GLMs assuming a small effect size of Cohen’s f^2^ = 0.10. With 160 subjects (after drop-out and exclusions) and with moderately correlated predictors (average r = .5) after multiple testing adjustments, we have well over 80 % power to detect effect with family-wise corrected alpha level of p < .05 [90].

## Discussion

The project aligns tightly with the goals and units of measurement defined by RDoC [10], to advance a neurobiologically informed understanding of mental disorders. Currently our understanding of the brain basis of anxiety and mood disorders is limited, especially at the individual patient level. To our knowledge, no previous study has characterized types of brain circuit dysfunctions across disorders and then evaluated how these dysfunctions relate to specific symptom profiles, behaviors and real-world functioning. Defining these types of dysfunction and relationships in a cohesive way will provide a foundation for ultimately tailoring interventions to individual needs.

We suggest that, to move forward in understanding the mechanisms of anxiety and depression and potentially improve quality of life, a shift is required to understanding the brain circuit domains linked to observed symptoms, behavior and function. Given that anxiety and depression are so prevalent [1, 2] generate significant disability [3] and we do not yet have biomarkers to help guide treatment decisions in clinical settings this study addresses an important public health need to understand these disorders from a neurobiological rather than diagnostic point of view -- in order to better explain the mechanisms by which dysfunction occurs and to better treat these disorders.

Strengths and innovation of this project include the use of mechanistically delineated paradigms for probing brain circuits and behavior such that brain-based constructs can be interpreted in the context of their behavioral expression as well as symptoms, the grounding in a conceptual model that goes beyond simply emotion processing to cognition and real world function, the use of well-established standardized assessments, and the clinical partnership that ensures recruitment of a sample representative of the community of people with anxiety and mood disorders.

## Limitations and challenges

We are conscious that recruitment of participants from community settings is a complex undertaking, which requires us to consider the clinical priorities of therapists and center supervisors. There is also a need to ensure a smooth flow for testing visits and to minimize participant burden. We have optimized the flow by using standardized assessments that minimize assessment time and by establishing a participant-friendly environment for the assessments. We also ensure that one research coordinator maintains contact with each participant to individualize the experience and to maximize retention in the study. Data collection will generate a large volume of data, and we have sought to address data management issues by establishing an efficient server infrastructure.

Despite these challenges, the project is expected to have an immediate impact on delineating the brain circuits and behavior that define cohesive constructs within the spectrum of anxiety and mood disorders, resulting in a new approach to diagnoses of anxiety and mood and provide a foundation for future tailoring of treatments to individual needs.

## Footnotes

1 The NIMH award title for this project is “Neural dimensions of threat reactivity and regulation for understanding anxiety”
